# Contraception for adolescents with lupus

**DOI:** 10.1186/1546-0096-8-10

**Published:** 2010-03-31

**Authors:** Melissa S Tesher, Amy Whitaker, Melissa Gilliam, Linda Wagner-Weiner, Karen B Onel

**Affiliations:** 1 Section of Pediatric Rheumatology, Department of Pediatrics, University of Chicago Medical Center, 5841 South Maryland Avenue, Room C101, MC 5044, Chicago, Illinois 60637, USA; 2 Department of Obstetrics and Gynecology, University of Chicago Medical Center, 5841 South Maryland Avenue, MC 2050, Chicago, Illinois 60637, USA

## Abstract

Sexually active adolescents, including young women with lupus, are at high risk for unplanned pregnancy. Unplanned pregnancy among teens with lupus is associated with an elevated risk of poor maternal and fetal outcomes. The provision of effective contraception is a crucial element of care for a sexually-active young woman with lupus. Unfortunately, providers may be hesitant to prescribe contraception to this group due to concerns about increasing the risk of lupus complications. This article reviews the risks and benefits of currently-available contraceptives for young women with lupus. Providers are encouraged to consider long-term, highly-effective contraception, such as implantables and intrauterine devices, for appropriately selected adolescents with lupus.

## Introduction

Systemic lupus erythematosus (SLE) is a chronic, multisystem autoimmune disorder which affects between 40 and 200 per 100,000 people in the United States and Europe, depending on ethnic background. Between 15-20% of these cases occur in children and adolescents [1-3]. The female-to-male ratio of affected patients is between 5:1 and 10:1[4]. Adolescent girls are therefore well-represented among patients with SLE. Appropriate counseling about contraception is an essential part of the care of any adolescent. Young people with lupus are typically evaluated more frequently by their pediatric rheumatologist than by their primary care provider, and in many cases the pediatric rheumatologist functions as the primary physician. Comfort with contraceptive counseling in general, and knowledge of the options available to adolescent girls with lupus in particular, are therefore of vital importance to the pediatric rheumatologist.

In 2000, the pregnancy rate among girls aged 15 to 19 in the United States was 84.5 per 1000, which represented a decrease compared to the 1980s and 1990s but still greatly exceeded rates of teen pregnancy in Canada and Western Europe. Of these pregnancies, almost 80% were unintended, and just over half resulted in a live birth [5]. Data from 2005-2007 show a trend toward increasing teen pregnancy in the United States[6]. Surveys of adolescent behavior show stable rates of sexual activity, but declining contraception use. These findings are potentially attributable to decreased intensity of national HIV-prevention efforts and an increasing emphasis on abstinence-only sex education curricula which do not provide contraception information [7]. Most women with SLE have normal fertility, and sexually active adolescent girls with lupus are thus at risk for unplanned conception [8]. For women with lupus, pregnancy poses particular risks, and practitioners must expend extra effort to help these patients avoid unplanned pregnancy. Risks during pregnancy for women with SLE include worsening disease, obstetric and fetal complications, and adverse effects of medications on fetal development.

## Pregnancy and fetal complications in women with SLE

Lupus flares may occur more frequently during pregnancy, although this remains controversial[9]. Disease status pre-conception is the most important factor impacting risk of flare during pregnancy, underscoring the need for women with lupus to plan their childbearing [10]. The assessment of lupus flare in pregnant women is complicated by several factors. First, no universally accepted definition of lupus flare exists. Many common signs of mild-to-moderate lupus flares, especially fatigue and musculoskeletal complaints, are frequently observed in normal pregnancy. Pregnancy can be associated with laboratory changes, including increases in ESR and C3, and classical complement activation with decreased C4, which complicate disease assessment[11]. Finally, preeclampsia may be difficult to differentiate from lupus nephritis[12]. Prospective studies of pregnancy in women with SLE have yielded conflicting results, but risk of flare appears elevated during the second trimester, third trimester, and puerperium in at least a subset of patients[13-15]. In particular, renal and hematologic flares may be more frequent, while musculoskeletal flares are decreased[16].

In addition to flares, obstetric complications and poor fetal outcomes are more common among women with SLE compared to healthy women. A review of women treated at the Hopkins Lupus Pregnancy Center from 1987 to 1996 found that hypertension, preeclampsia, hyperglycemia, gestational diabetes, and bladder infections were more frequent in pregnant women with lupus. With the exception of bladder infections, all complications were more frequent in women taking more than 10 mg/day of prednisone. It is unclear whether these effects are primarily due to underlying active disease requiring more intensive prednisone therapy, or to prednisone's known adverse effects on blood glucose levels and blood pressure. The Hopkins Lupus Pregnancy Center series also reported a significant increase in preterm birth, most frequently caused by premature rupture of membranes. The incidence of intrauterine growth retardation (IUGR) was also increased, was more marked among African-American infants than among white infants, and tended to be more dramatic the longer the gestational period. Patients with positive antiphospholipid antibodies experienced frequent pregnancy loss, especially at 20 weeks gestation or later. Lupus anticoagulant was a much better predictor of fetal loss than were anticardiolipin antibodies. Neonatal lupus syndrome, an uncommon but serious complication of lupus pregnancy, is strongly associated with maternal anti-Ro antibodies and can lead to complete heart block. No infant in this series was affected by neonatal lupus syndrome, reflecting the rarity of this entity [16]. A 2008 review of data from the National Inpatient Sample evaluated available information on over 13,000 admissions for lupus childbirth. As in the Hopkins series, women with SLE were at significantly increased risk for pregnancy loss, preterm delivery, preeclampsia, maternal infections, and thrombosis [17].

More recent series of lupus pregnancies have elucidated particular risk factors for adverse maternal and fetal outcomes. A retrospective review was performed of 58 SLE patients with 90 pregnancies, seen at the Mayo Clinic between 1976 and August 2007. Active lupus nephritis (defined as active urinary sediment with >0.5 g/day of proteinuria, with normal or abnormal serum creatinine) predisposed to fetal loss, premature delivery, and obstetric complications including hemolysis, elevated liver enzymes and low platelets (HELLP) syndrome, hypertension, and preeclampsia, as compared to women with SLE but without lupus nephritis. Quiescent lupus nephritis was not associated with any additional pregnancy risks compared to women with SLE who had never experienced nephritis [18].

Even when disease is inactive, adjustments to previous treatment regimens are likely to be necessary to optimize pregnancy outcome. Many medications used in the treatment of lupus are known teratogens or abortifacients. Mycophenolate mofetil is often used for lupus nephritis therapy, and as a steroid-sparing agent for other manifestations of SLE. In animal studies, mycophenolate mofetil caused developmental abnormalities. Multiple human cases of major fetal abnormalities including facial clefts and ear anomalies have been reported. Mycophenolate is classed in pregnancy category D (positive evidence of human fetal risk) [19]. Methotrexate, used to treat cutaneous and musculoskeletal involvement and as a steroid-sparing agent, is a well- known abortifacient which is often utilized to treat ectopic pregnancy [20]. Methotrexate has also caused severe congenital malformations including orthopedic, facial, skull, and central nervous system abnormalities [21]. Cyclophosphamide, an alkylating agent used to treat renal and central nervous system complications of lupus, is associated with palate, limb, eye, and skeletal malformations in humans when used in the first trimester[22,23]. Thalidomide, occasionally prescribed to treat severe cutaneous lupus, is a notorious teratogen causing severe limb deformities[24].

Beyond the pregnancy risks confronted by all women with lupus, adolescents with SLE face additional risks associated with young age of childbearing. Teenage mothers in general are at increased risk for premature delivery, low birth weight or small for gestational age infants, and neonatal mortality, compared to mothers ages 20-24 years. Pregnant adolescents are more likely than adult women to be poor, unmarried, to receive inadequate prenatal care, and to have low levels of education. However, two large studies showed that relative risks for adverse neonatal outcomes persisted even after controlling for these socioeconomic and demographic factors[25,26].

## Contraception

Effective contraception is crucial for sexually active adolescents with SLE, given the multiple risks of pregnancy, and the complex medical management required to optimize outcomes. Frank, open discussion with adolescent patients with SLE regarding their sexual activity is an important part of patient care. Rheumatologists must emphasize the importance of protection from sexually transmitted infections (STIs) and unplanned pregnancy, should the patient decide to become sexually active. No contraceptive method is ideal; all have benefits and drawbacks. The risk of use of a given contraceptive must be balanced against the known significant risks of pregnancy in an adolescent with lupus. The lack of definitive guidelines should not impede the provision of effective contraception to this very high-risk group.

Male and female condoms are safe, inexpensive, and, when used consistently and correctly, prevent both pregnancy and transmission of STIs. Unfortunately, consistent condom use is rare among adolescents. Fewer than 40% of sexually active male and female respondents under age twenty years reported using condoms at last intercourse[27]. Increased use correlates with patient beliefs that condoms are popular with peers, easy to obtain, build relationship trust, and promote partner responsibility. In addition, high perceived self-efficacy at obtaining condoms and negotiating for their use increases condom use[28,29]. Practitioners caring for sexually active adolescents with SLE may increase the effectiveness of their counseling by emphasizing the aforementioned health beliefs, in addition to discussing STI and pregnancy prevention. Despite all of the above, the first-year typical-use contraceptive failure rate of condoms, about 15%, (21% for the female condom) is unacceptable in a high-risk population. Barrier methods other than condoms, such as the diaphragm and cervical cap, do not protect against STIs and have unacceptably high typical use failure rates (16% to 31%, depending on the method chosen and parity of the user) [30]. While rheumatologists should encourage condom use by sexually-active patients to protect against STIs, a second contraceptive method should also be recommended for highly effective pregnancy protection.

Research on safety of non-barrier contraceptive methods in women with SLE is scarce. Physicians must select a contraceptive method on a case-by-case basis, with the active participation of the adolescent patient. Frequently the best choice is determined by which method the patient is willing to use consistently. Methods to consider include combined estrogen and progestin contraceptives including oral contraceptive pills(OCPs), contraceptive patches, and vaginal rings; progestin-only methods including injectables, implants, and progestin-only pills; and intrauterine devices. Sexually active adolescents should be referred for gynecological examination as part of their general health maintenance. However, current evidence does not support mandatory pelvic examination prior to prescribing hormonal contraception[31].

Despite the popularity of estrogen-containing combined contraceptives, many practitioners are hesitant to prescribe these to women with SLE due to concerns that exogenous estrogens could provoke lupus flares[32]. SLE most often occurs in post-pubertal women, and as discussed above, flares may be increased with pregnancy, lending credence to the hypothesis that SLE disease activity can be augmented by estrogens. Various autoimmune effects of estrogen have been observed in vitro, including enhanced T-cell resistance to apoptosis, and increased numbers of pathogenic naïve autoreactive B cells. Increased estrogen has also caused enhanced autoimmunity in murine models of SLE[33]. However, the clinical applicability of these findings is uncertain.

Despite the completion of several well-designed studies, the safety profile of estrogen-containing oral contraceptives in women with lupus is not clear. Sanchez-Guerrero et al, in a 2005 study, randomly assigned 162 Mexican women with SLE to combined oral contraceptives, a progestin-only pill, or a copper IUD. Women with highly active disease (SLEDAI >30) at baseline or a history of thrombosis were excluded. Interestingly, women with positive antiphospholipid antibodies and smokers (except women over 35 who smoked >15 cigarettes/day) were included. The investigators found no significant differences between groups in incidence of flares or global disease activity. There were four occurrences of serious thromboses, two each in the OCPs and progestin-only groups, all in women with low-titer positive antiphospholipid antibodies[34]. While these data suggest that OCPs are safe for at least some patients with SLE, this study was limited by the large number of patients excluded (of 1981 patients screened, 1533 did not meet inclusion criteria), as well as by the inclusion of only one ethnic group. Also in 2005, the SELENA-OC study, a multicenter randomized, placebo-controlled trial of combined oral contraceptives in 183 women with lupus, showed no significant difference in disease activity over one year. This study included patients with inactive or stable disease, and excluded women with hypertension, those requiring high (>0.5 mg/kg/day) oral steroids, and those with evidence of antiphospholipid antibodies (lupus anticoagulant or high-titer anticardiolipins), among others. Disease flares and serious complications including thrombosis and infection were similar between groups[35]. The SELENA-OC study results are reassuring regarding safety of OCPs for a subgroup of women with SLE who are normotensive with mild, stable disease and no known thrombotic risk factors, but again, relatively few women with SLE meet these conditions. Studies of exogenous estrogens in women with severe SLE with major organ involvement have not been performed.

Oral contraceptives do have significant benefits, including high effectiveness when used correctly, relatively low cost, and the ability to discontinue use at any time. Unfortunately among adolescents, poor compliance poses a significant barrier to effective use of oral contraceptives[36]. Up to 20-30% of adolescents miss at least one pill per month. Recommendations on combined oral contraception for women with SLE can likely be extrapolated to non-oral forms of combined hormonal contraception such as the contraceptive patch and the vaginal ring, although specific studies are lacking. The vaginal ring (marketed in the United States as the NuvaRing), in particular, offers some advantages. The NuvaRing releases 15 μg per day of ethinyl estradiol, less than low-estrogen oral contraceptives, and frees the woman from the need to remember a daily pill. A large international survey of NuvaRing users in 2002 showed high continuation and user satisfaction rates, although it did not specifically address adolescents[37].

Unfortunately, estrogen-containing contraceptives increase the risk of venous thromboembolism, and are not recommended for patients with lupus anticoagulant, antiphospholipid antibodies or history of thrombus[38]. Overall, young women with lupus who are responsible, motivated nonsmokers, with negative antiphospholipid antibodies, no history of thrombosis, and stable disease without renal involvement, may be considered for combined contraceptive therapy. To minimize the risk of thrombotic complications, a low-estrogen formulation should be selected, and the patient should be warned to avoid tobacco use. The majority of adolescents with lupus are likely not good candidates for estrogen-containing contraception, and alternatives should be explored.

Several non-estrogen-containing hormonal contraceptives are available. Studies specifically addressing thrombotic risk of progestin-only methods, particularly in high risk populations, are not available. However, considering the known thrombotic risks of estrogen-containing contraception, non-estrogen methods are likely a more prudent choice in patients with other prothrombotic risk factors. The progestin-only oral contraceptive ("minipill") must be taken at the same time each day to maximize efficacy, raising concerns about adherence in adolescents. Menstrual irregularities are frequent, leading to a discontinuation rate above 50% in some studies[39]. In a 1989-90 survey of 85 Finnish women with SLE who were of reproductive age, 32 women (38%) had previously used progestin-only oral contraceptives. Seventy-eight percent of them, however, had discontinued this method due to side effects[40].

Depot medroxyprogesterone acetate (DMPA) injections, another progestin-only method, provide easy to use, highly-effective contraception. Injections are needed only every three months, and DMPA is readily available and inexpensive. However, long-term use is associated with decreased bone mineral density (BMD)[41]. While the decrease in BMD may not be clinically significant in healthy adolescents, it may be of concern for women with SLE who have an increased risk of osteopenia due to disease and to use of corticosteroids. Calcium and vitamin D supplementation is prudent for adolescents with lupus who utilize DMPA [42,43]. Irregular menses and weight gain are also frequent with DMPA use, and continuation among adolescents is poor[44]. Implanon, a single-rod etonogestrel-releasing implantable contraceptive, is relatively new, and has several attractive features. Implanon offers highly-effective contraception for up to three years, is rapidly reversible, and is easily placed and removed. As with other progestin-only methods, menstrual irregularities are frequent[45]. Unlike DMPA, Implanon does not appear to have a detrimental effect on bone density. A 2000 study evaluated BMD over two years of use of Implanon, compared to women using a non-hormonal IUD, and found no significant difference[46]. Although data on continuation of Implanon in adolescents are lacking, this method could be an excellent choice for some young women with SLE.

Young women with SLE who desire long-term contraception may also consider the intrauterine device (IUD). IUDs offer many potential advantages to women with SLE. They are highly effective, long-lasting, and easily reversible. IUDs are generally underused in the United States, mainly due to concerns of increased susceptibility to pelvic inflammatory disease (PID) and subsequent risk of decreased fertility. However, an increasing body of evidence suggests that these concerns may be exaggerated. A large World Health Organization survey reported that among 4031 women in China who had IUDs inserted, no case of PID occurred in 9197 woman-years of observation. In Africa, where STDs are more prevalent than in China, only eight cases of PID occurred during 1292 woman-years of follow-up. The period of increased risk was limited to the first 20 days post-insertion[47].

Specific studies of IUD safety in women with lupus are limited. In Julkunen's 1989-90 cross-sectional survey of women with SLE in Finland, forty-four women reported current or previous IUD use, and none had experienced a major infection or bleeding complication[41]. Sanchez-Guerrero's 2005 study compared progestin-only oral contraceptives, combined oral contraceptives, and the copper IUD over a one-year period in 54 women with SLE. Four women experienced expulsion of the IUD. Five serious infections occurred in the IUD group, a non-significant increase compared to the other groups. The infection risk attributable to IUDs is difficult to determine, as two of the infections were meningitis, and the authors do not specify whether the other serious infections were of pelvic origin[35]. However, data on IUD infection risk in other immunosuppressed populations, specifically HIV-infected women, are encouraging. Rates of PID in IUD users were not significantly increased, even among severely immunocompromised women, compared to HIV-infected women using other contraception or no contraception [48,49].

The copper IUD frequently increases menstrual blood loss, sometimes causing anemia. Dysmenorrhea is also frequent. The possibility of IUD expulsion may be higher in young, nulliparous women, and patients should be educated regarding signs of device expulsion [50]. The levonorgestrel-releasing intrauterine system (LNG-IUS, marketed in the U.S. as Mirena) may be a better alternative than the copper IUD for adolescents because of its potentially reduced risk of PID compared to the copper IUD and cycle control benefits [51,52]. Amenorrhea frequently occurs with use of the LNG-IUS, and this device has been successfully used for treatment of menorrhagia[53]. Specific studies of thromboembolic risk of levonorgestrel alone are lacking. However, levonorgestrel-containing combined oral contraception appeared in some studies to have reduced thrombophilic potential as compared to OCPs containing "third-generation" progesterones such as desogestrel[54]. Although data on IUD use in adolescents is limited, available evidence suggests high continuation rates, comparable to or better than rates for OCPs[51].

If unprotected sex occurs, due to either contraceptive nonuse or method failure, emergency contraception (EC) can reduce the likelihood of unintended pregnancy. Emergency contraception in the form of oral levonorgestrel has recently become available without a prescription in the United States, for purchasers age 17 and over. As discussed in the preceding paragraph, available evidence indicates a low risk of thrombosis for oral levonorgestrel. Moreover, the 2005 American College of Obstetrics and Gynecology policy statement on EC reported that no deaths or serious complications attributable to EC have occurred, and recommended that EC be made available to all women including those with contraindications to OCPs [55].

## Conclusions

Clearly, sexually-active adolescents with SLE require highly-effective contraception with minimal adverse effects, which can represent a clinical challenge. Research specifically addressing contraceptive issues in women with SLE is limited. However, considering the risks to the adolescent patient with SLE of an unplanned pregnancy, providers must not be deterred from providing effective contraception. Although condom use is important and should be encouraged, it is not adequate to prevent pregnancy in this high-risk population. A combined oral contraceptive may be appropriate for a subset of adolescents with good adherence, mild-to-moderate disease activity, and no evidence of hypercoagulability. The progesterone-only minipill is unlikely to worsen lupus activity or promote thrombosis, but has high discontinuation rates. DMPA, which offers the advantage of greater typical-use efficacy than progesterone-only pills, is a reasonable choice for many adolescents. However, menstrual irregularities are frequent. DMPA's detrimental effects on bone density are also of concern. The etonogestrel-releasing implant appears to be a good choice for progesterone-only long-term contraception, although specific studies in women with lupus are lacking. Existing evidence does not support the opinion that a diagnosis of lupus, or immunocompromise, are contraindications to the use of an IUD. The copper IUD offers a non-hormonal alternative for long-term contraception. However, menorrhagia is frequent among users of copper IUDs, and as anemia is common in adolescents with SLE, the LNG-IUS may be a better choice. Adolescents and young women with lupus who choose to use an IUD should be educated about the slightly increased risk of upper genital tract infection in the first month after insertion, and should also be instructed on self-exams to check for unrecognized IUD expulsions. Education regarding the safety and availability of emergency contraception, and provision of an EC prescription to fill in case of need, are also appropriate in many circumstances. Regardless of the method chosen, active participation by pediatric rheumatologists in contraceptive counseling for adolescents with lupus is a critical part of these patients' care.

## Competing interests

The authors declare that they have no competing interests.

## Authors' contributions

KO, MG, and MT conceived of the project, MT and AW performed the literature review, MT drafted the manuscript, LW, KO, and AW participated substantially in writing and revisions.
